# Neurophysiological Correlates of Concussion: Deep Learning for Clinical Assessment

**DOI:** 10.1038/s41598-019-53751-9

**Published:** 2019-11-22

**Authors:** Rober Boshra, Kyle I. Ruiter, Carol DeMatteo, James P. Reilly, John F. Connolly

**Affiliations:** 1 ARiEAL Research Centre, McMaster University, Hamilton, Canada; 2 School of Biomedical Engineering, McMaster University, Hamilton, Canada; 3 Vector Institute, MaRS Centre, Toronto, Canada; 4 Linguistics and Languages, McMaster University, Hamilton, Canada; 5 School of Rehabilitation Sciences, McMaster University, Hamilton, Canada; 6 Electrical and Computer Engineering, McMaster University, Hamilton, Canada; 7 Psychology, Neuroscience & Behaviour, McMaster University, Hamilton, Canada

**Keywords:** Health care, Neurological manifestations, Brain injuries

## Abstract

Concussion has been shown to leave the afflicted with significant cognitive and neurobehavioural deficits. The persistence of these deficits and their link to neurophysiological indices of cognition, as measured by event-related potentials (ERP) using electroencephalography (EEG), remains restricted to population level analyses that limit their utility in the clinical setting. In the present paper, a convolutional neural network is extended to capitalize on characteristics specific to EEG/ERP data in order to assess for post-concussive effects. An aggregated measure of single-trial performance was able to classify accurately (85%) between 26 acutely to post-acutely concussed participants and 28 healthy controls in a stratified 10-fold cross-validation design. Additionally, the model was evaluated in a longitudinal subsample of the concussed group to indicate a dissociation between the progression of EEG/ERP and that of self-reported inventories. Concordant with a number of previous studies, symptomatology was found to be uncorrelated to EEG/ERP results as assessed with the proposed models. Our results form a first-step towards the clinical integration of neurophysiological results in concussion management and motivate a multi-site validation study for a concussion assessment tool in acute and post-acute cases.

## Introduction

Traumatic brain injury (TBI) impacts upwards of 2.8 million individuals annually in the united states alone^1^. Concussions (henceforth used synonymously with mild TBI; mTBI) form a considerable subset of that figure and are defined as closed-head injuries that leave the affected with functional and cognitive deficits^2,3^. The current understanding of underlying mechanisms in concussion remains lacking, with echoing concerns both in the identification and management of the condition^4^. An expansive body of work has targeted the multiple facets of concussion, offering different means of elucidating the cognitive deficits caused by concussion and its co-morbid sequelae^5^. Electrophysiology is one tool with promising applications in concussions. Specifically, event-related potentials (ERPs) as recorded by electroencephalography (EEG) have shown persistent changes in concussed individuals in the post-acute stage and decades after insult^6–10^.

ERPs are non-invasively-recorded indices of cognitive function^11^. The P300, a positive-deflecting response peaking approximately 300 ms after stimulus onset, is a commonly studied component in neurophysiology that is associated with attentional resource allocation, orientation, and memory^12^. The P300 was found to be impacted by concussion immediately^13^ after occurrence and decades post injury^6,8–10^. P300 effects were observable when patients were symptomatic as well as after symptom resolution^14^ and were affected cumulatively following a series of concussive blows to the head in comparison to a single hit^15^. The N2b is an ERP often linked to executive function manifesting as a fronto-central negative deflection 200 ms after stimulus onset^16^. Similar to the P300, the N2b was affected after sustaining hits to the head^7,10,15,17,18^. Research has demonstrated the versatility and sensitivity of both the P300 and N2b to concussion; however, a transition from controlled, group-level findings to individual assessment is required before clinical adoption is made feasible.

Machine learning (ML) has gained significant traction in the clinical field, offering a cost-efficient way of replicating expert judgements and decisions in a setting overloaded with data^19,20^. ML introduces a dynamic process that is able to ingest high-dimensional clinical data and learn complex patterns that might also be difficult to detect or visualize for a human expert^19,20^. Despite some scrutiny due to black-box solutions^21^ and susceptibility to bias in misapplication^22^, machine learning remains a great tool for exploiting resources to improve clinical standards^19,21–23^. EEG data are characterized by their rich high-dimensionality that requires certain degrees of aggregation to simplify for a human observer – quite possibly at the cost of losing critical information. That complexity has made ML a valuable method in EEG analysis^24–32^.

Although this study details the first EEG/ERP application of deep learning (DL) in mTBI, DL has been explored in various EEG applications^33^. Broadly, DL expands on traditional ML techniques by providing a multi-layer architecture that enables fitting complex and custom models that promote hierarchical feature extraction. In EEG, model complexity and layer stacking has been proposed as a valuable tool in creating end-to-end solutions that integrate feature extraction and classification as opposed to the more manual feature engineering of traditional ML^24^. Most DL applications on EEG to date have been on resting-state, using shifting windows in time as input, to provide datasets with sufficient size for training such complex models^27,28,33^. Recently, there have also been studies of DL to classify targets (P300) vs. non-targets in a brain-computer interfacing setup^27,33^.

**Figure 1:**
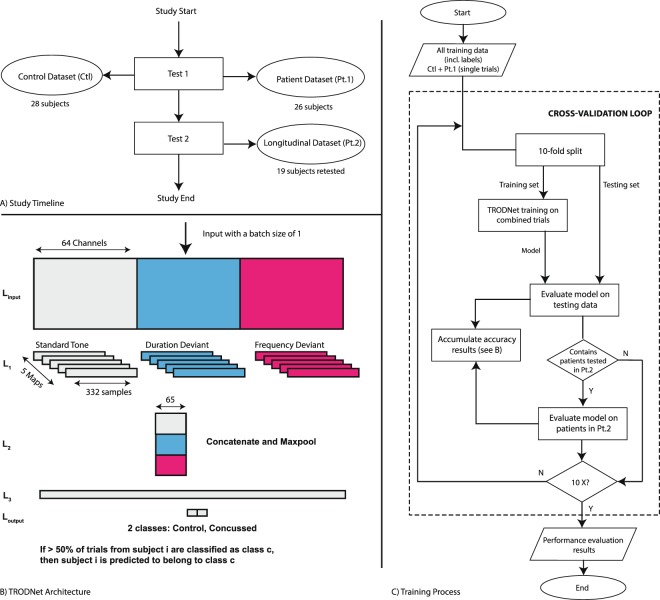
(**A**) The general study timeline and datapoints collected from each group. (**B**) The TRODNet architecture and sizes provided an input batch of size 1 that contains data from 64 channels and 3 experimental conditions across 332 samples. (**C**) The training/testing procedure accounting for both concussed subsets.

In the present study, we developed the TRauma ODdball Net (TRODNet), a deep learning network that uses convolutional layers in extracting information from single-trial EEG/ERP data to identify signs of concussion. The network learns a set of topographical maps that characterize different ERPs elicited in a multi-deviant oddball paradigm designed to elicit both the P300 and the N2b responses. The temporal activations of these maps form a set of automatically extracted features to predict a single-trial’s label. TRODNet is trained and assessed using 10-fold class-stratified cross-validation on a dataset of 54 participants (28 controls). All concussed participants were clinically diagnosed and were symptomatic at the time of testing. Supplementary self-reports were collected to investigate concussive and depressive symptomatology as captured by the post-concussion symptom scale (PCSS) and the Children Depression Inventory 2 (CDI), respectively. Nineteen of the 26 concussed subjects returned for a follow-up test (see Fig. 1A), nine of which reported full symptom recovery (PCSS of 0) with the others developing post concussion syndrome (PCS). Analyses on the longitudinal samples were run in parallel to assess whether symptom resolution was identifiable by the trained model (see Fig. 1C). Model interpretation is a critical factor for integrating machine learning into the clinical setting^21^. Thus, trained models were interpreted using the SHapley Additive exPlanations (SHAP) method, a recent introduction to the field with demonstrated success in clinical applications^23,24,34^.

The study was designed to investigate two primary hypotheses. First, the study examined whether single-trial classification can be aggregated for each subject to provide a viable tool of detecting concussion-related neurophysiological effects using minimal feature engineering. Second, the model’s judgements on longitudinal datapoints were examined. It was postulated that performance would deteriorate after symptom resolution due to a normalization of the recovered subjects’ neurophysiological responses, as opposed to consistent performance in those who retained their symptoms. Model interpretability was prioritized to ensure a transparent representation of learned information and to serve as a confirmatory step for the model’s results.

## Results

### Concussion identification

As the model was trained (and tested) on single trials, aggregation of the TRODNet output was performed to create a prediction on the subject-level (see Methods for more details). As such, if more than 50% of a subject’s trials were classified as concussed, the subject was predicted as belonging to the concussed group. The TRODNet model was able to achieve a single-subject cross-validation accuracy of 85%. Specifically, four control subjects were misidentified as concussed while four concussed subjects were misclassified as controls. This put the model’s sensitivity to concussive effects at 84.6% and its specificity at 85.7%. Single-trial cross-validation accuracy was recorded at 74.4%; however, this figure should be assessed with care as discussed below. A detailed list of the model’s single-trial accuracies; PCSS and CDI scores; demographics; and number of days since injury for each subject in the concussed group, including the longitudinal results, is reported in Table 1

**Table 1:** Table detailing the symptomatology and depression scores for all concussed participants.

ID	Sex	#Prev Concs	#Days since injury	PCSS	CDI	Accuracy
1	F	6	36	109	71	94.4
**2**	F	0	20–225	55–54	68–59	72.2–41.7
**3**	F	1	5–121	54–17	68–68	86.1–63.9
4*	M	2	23–86	20–0	76–40	77.8–61.1
5	M	2	30	64	49	86.1
6	M	2	7	33	57	5.6
7*	F	2	14–139	35–0	46–40	97.2–86.1
8	F	6	8	94	52	66.7
**9**	F	1	17–79	92–6	67–43	66.7–58.3
10*	F	1	9–211	67–0	51–41	97.2–97.2
11*	F	1	17–163	50–0	46–42	52.8–47.2
**12**	M	5	14–104	101–54	63–54	69.4–58.3
13	F	1	13	41	43	72.2
**14**	F	4	15–240	24–24	58–63	61.1–58.3
**15**	F	3	30–135	58–11	46–47	75.0–30.6
16*	F	0	7–98	17–0	43–40	94.4–88.9
17*	F	1	8–85	12–0	47–40	80.6–77.8
**18**	M	2	19–187	46–31	44–49	61.1–69.4
**19**	F	1	12–180	53–20	62–66	8.3–22.2
20	M	1	58	55	49	36.1
**21**	F	0	30–172	59–82	68–76	50.0–5.6
22	F	2	39	60	71	66.7
**23**	F	2	26–174	80–46	67–55	97.2–91.7
24*	F	1	6–181	55–0	63–42	72.2–55.6
25*	M	1	13–113	32–0	46–49	100–94.4
**26**	F	1	48–118	66–3	55–52	88.9–91.7
**Concussed subjects:**						
-First test, all	19 F, 7 M	1.9 (1.6)	20.2 (13.6)	55.1 (25.4)	57 (10.5)	70.6 (24.8)
-Second test, SR	6 F, 2 M	1.1 (0.6)	134.5 (47.0)	0	41.8 (3.1)	76 (19)
-Second test, NSR	9 F, 2 M	1.8 (1.6)	157.7 (50.6)	31.6 (24.6)	57.5 (10)	53.8 (27)
-Second test, all	15 F, 4 M	1.5 (1.3)	147.9 (49.2)	18.3 (24.4)	51 (11.1)	63.2 (25.9)
**Controls**	23 F, 5 M	0	NA	NA	NA	77.4 (20.8)

Bolded subject IDs (19) represent the ones who returned for a follow-up EEG test. Where applicable, second testing (t2) values are presented after the first (t1) values as in: t1, t2. Asterisks (*) on subject IDs denote the concussed subgroup that reported full symptom recovery (PCSS of 0) by their second assessment. Aggregates for the concussed and control groups are presented, as well as for the longitudinal subsets.

### Longitudinal factors

**Figure 2:**
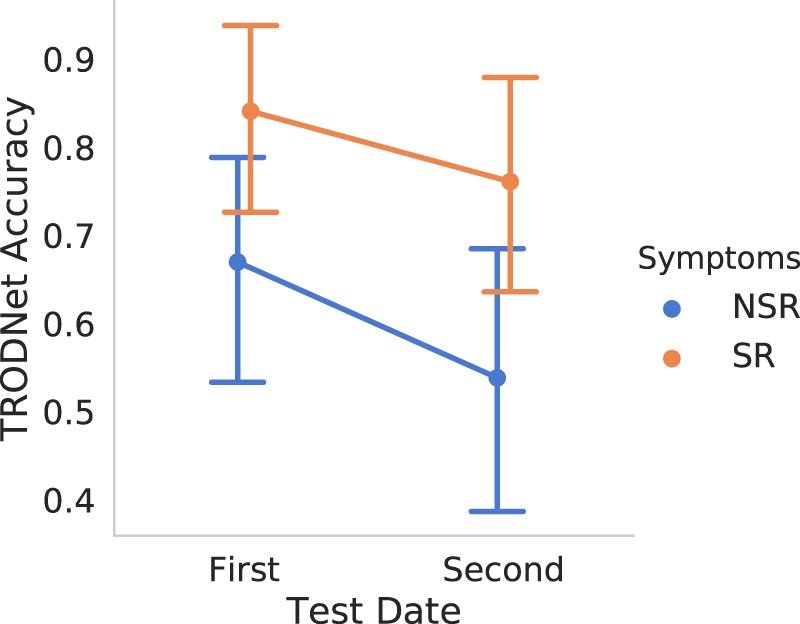
The interaction effect of Recovery and Testing Date on the TRODNet results as seen on the longitudinal subgroup. While there were main effects of both factors, no reliable interaction was found. Points represent mean prediction from TRODNet’s result, where 0 (1) is a classification of control (concussed). Vertical extended lines indicate the 95% confidence intervals.

Assessing the model’s single-trial accuracy for the concussed subgroup that participated in the follow-up test yielded a significant drop in accuracy (F(1,17) = 8.93, p < 0.01) in the second test compared to the first. A significant main effect of Recovery (symptom resolution [SR] vs. no symptom resolution [NSR]) was also found (F(1,17) = 4.84, p = 0.04), indicating a lower accuracy for the NSR group. Lastly, no significant Recovery × Testing Date interaction was observed (F(1,17) = 0.17, p = 0.69). Overall, the model assessed 14 of the 19 subjects as concussed at the second testing date. The interaction plot is presented in Fig. 2, showing a clear main effect of Testing Date that is not influenced by Recovery. Additionally, it can be observed that subjects that didn’t report symptom recovery had lower single-trial accuracies overall.

### Injury acuteness and correlation analyses

**Figure 3:**
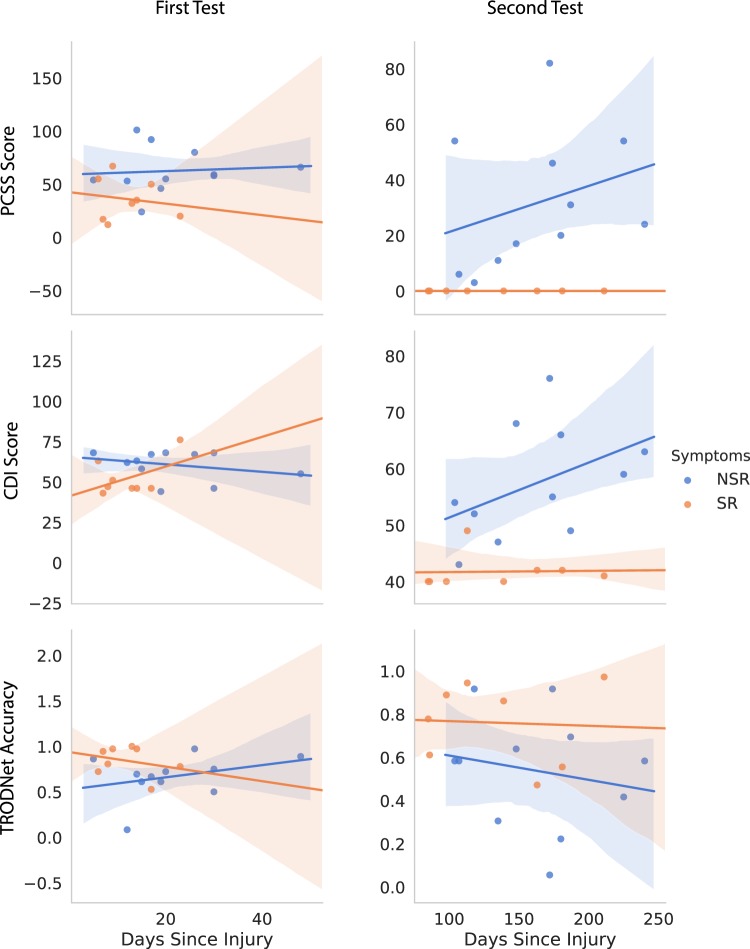
Interactions between days since injury and symptomatology (first row), depressive symptoms (second row), and TRODNet single-trial results in the longitudinal sample of our presented dataset (third row). The symptom resolution (SR) subgroup conveyed no identifiable patterns both in the first (left column) and second (right column) tests. The subgroup that did not have symptoms resolve (NSR) showed an increase in symptomatology and depressive signs as days since injury increased for the second test. Shaded regions signify the 95% confidence intervals.

The effect of days since injury on perceived results was inconclusive for the first day of assessment (see Fig. 3 and Table 1). For the second date, self-reported symptoms seemed to increase as days since injury increased for the no symptom resolution (NSR) group. This effect was equally observable in the PCSS and CDI scores. Although the two measures are inherently confounded, this result proposes a layer of subjectivity indicating a worsening of effects as an individual is subjected to symptom persistence. Conversely, no clear effect of days since injury was noted on the EEG/ERP results when accounting for symptom resolution. Overall, the SR subgroup reported a lower PCSS score at the date of the first test compared to the NSR subgroup. This is concordant with reports of symptom severity being a consistent measure of clinical recovery^2^.

### Insights from model explanations

**Figure 4:**
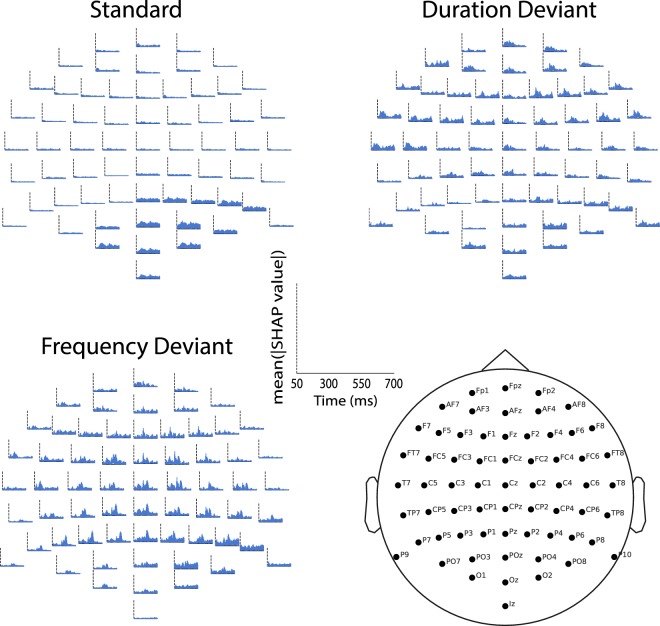
The mean of the absolute SHAP values for single-subject averages overlaid on the head for each condition and electrode. The abscissa denote time where 0 is the stimulus onset. The ordinate represents the mean absolute SHAP value at the indicated electrode, time, and condition. The figure shows a robust identification of ERPs of interest, particularly in the frequency (FDev) and Duration (DDev) deviants. An interesting effect can be observed to the standard condition where the parieto-occipital region has a widespread effect predominantly in the right hemisphere.

Upon interpreting the model with SHAP, TRODNet highlighted areas of interest overlapping with previously demonstrated effects in the literature^10,35^. The mean absolute SHAP values, indicative of feature importance, were reshaped for display on a 64-channel EEG plot for each condition (see Fig. 4). The two deviants had the most prominent features with important ones forming a bimodal distribution in the posterior regions, morphing into a unimodal shape in the frontal areas. The first and second peaks correspond in time and topography to the P300 and N2b, respectively^12,16^. Features tended to be uniformly important bilaterally, with slightly higher importance for the right side. Responses to the standard condition showed smaller and more dispersed distributions of feature importance, an unexpected finding considering an earlier study on chronic effects of concussion that showed early discernible effects to the standard tones^24^.

## Discussion

Our results demonstrated the efficacy of an acute/post-acute automated system for concussion identification in individual subjects. In contrast to earlier work in concussion, the utilization of deep learning and convolutional networks enabled an end-to-end solution with minimal feature-engineering^24,26,36,37^. Additionally, the hypothesis that single-trials offer a more granular and effective method of assessing EEG/ERP data was supported.

Results relating symptomatology and neurophysiological effects were negative. Despite the misalignment between the present study’s hypothesis and the data, symptomatology has been previously shown to have little correlation to EEG/ERP effects^6,10,35^, especially as neuropsychological measures completely return to baseline in most cases^38^. This disagreement extends to other assessment modalities such as quantitative EEG^36,39^. It is noteworthy that the model’s performance drop may be attributable to the time-elapsed since injury, a finding that agrees with a regression study conducted in parallel to the present one (under review). These results highlight the need to examine the multiple stages of concussion progression and their effects with care as some may potentially be observable strictly at a particular stage of injury and/or recovery. Moreover, in the longitudinal subset, the model predicted trials of subjects that exhibited symptom resolution as concussed more than the subjects with persisting symptoms. Interestingly, that difference was observed irrespective of Testing Date (1^*st*^ vs. 2^*nd*^; Fig. 2). These results introduce the possibility that a subject’s recovery trajectory may be inferred from a participant’s EEG/ERP results during their symptomatic stage; however, no strong evidence could be drawn given the constraints of the present dataset. Of note, performance in the longitudinal sample is difficult to interpret provided that at no time was the model trained on a longitudinal sample from our data. We are not able to draw conclusions on whether the results are due to PCS-related neurophysiology or a more broad neurophysiological persistence of the injury that remains beyond symptom resolution. In practical terms, we posit that a sufficiently-large PCS group is required in addition to a symptomatically-resolved group to train a model to effectively differentiate the two against a control group. Ideally, given sufficient data, a model should also have access to date from injury to properly factor for a dynamically changing manifold of injury-affected responses.

The present study is the first report of ML-based EEG/ERP analysis in acute/post-acute concussion assessment. We reported a higher accuracy than previous studies classifying mTBI using RS EEG^26,37^ and marginally higher than a previous study on injury detection decades after injury^24^. A quantitative comparison with clinical tools typically used in mTBI assessment is not straightforward as some of the best-reported tools decline in utility as soon as 5 days after injury^2,4^ while our first day of data collection was an average of 20.2(13.6) days separated from injury. Clinical tools such as self-reported symptoms, postural control evaluation, and a pen-and-paper assessment scored sensitivities of 68.0%, 61.9%, and 43.5%, respectively, when administered within 24 hours of injury^40^. Combining all these tools was reported to exceed 90% sensitivity, although it is critical to be mindful that with these increments in sensitivity, specificity of these methods deteriorates and, by definition, reduces accuracy. Overall, we argue that the implementation of a single-subject EEG/ERP evaluation for acute/post-acute concussion is feasible provided group-level studies in the literature^17,35^ and extended to single-subjects by the methodology presented here. Clinical applicability beyond the acute stage, however, requires further investigations that would augment the data used for training as discussed above.

The interpretability layer on our neural network model confirmed our results’ origins as pertaining to neurophysiological signals commonly affected by concussion. This provides strong evidence that the model’s predictive power is linked to the ERPs that the experimental paradigm was designed to elicit. Primarily, in the deviant conditions, TRODNet’s most important features, as extracted by SHAP, corresponded to the 100–500 ms window, encompassing both the N2b and the P300 (see Fig. 4). Topographical examination of feature importance showed the effects to be predominantly central, with an earlier effect that is marginally lateralized to the right. Examination of the standard condition showed a small parieto-occipital effect in the 100–300 ms range, likely related to the N1-P2 complex. While this finding agrees with previous work on chronic neurophysiological effects of concussion observable in responses to the standard tones in an oddball paradigm, the features show low and dispersed importance measures compared to what was observed in the earlier study^24^. This is compatible with a hypothesis that alterations in earlier responses (in the mismatch negativity^10^ or the N1/P2 complex^24^) may correspond to irreversible effects of concussion and are strictly prominent in chronic cases. Further, tracing the model’s results provides additional, empirical and data-driven, support of mTBI’s impact on facets of cognitive function linked to the P300 and N2b such as attention and executive function^10,17,41^.

The study exhibits two primary limitations. First, the difference in age between the two groups can be argued to contribute to the model’s ability to discern between the two experimental groups. Although there have been several reports of age-related differences in ERPs and resting-state EEG, the evidence supports little to no differences in the range of our two groups (15.04 and 19.3)^42–44^. Thus, we argue that an effect pertaining to the presented age-range is minimal, if not unlikely. Secondly, as correlations between model output and symptomatology were conducted post-hoc, further work is required to confirm the relationships between time-elapsed since injury and ERP effects.

In sum, a strong case for the clinical utility of ERPs in individual assessment of acute/post-acute concussion patients has been presented. The current findings improve upon those from resting-state and quantitative EEG^36,37^ to establish a modality that is able to capture the effects of concussion immediately after insult and years post-injury^24^. The intent of this research was not directed at the mechanisms of progression and symptom manifestation, which remain unclear. However, a major step in that direction has been achieved in the translation of a complex, multi-trial EEG signal that was successfully able to provide an accurate identification of concussion incidence on a single-subject basis. The proposed model, TRODNet, was able to capture distinguishing features without the need for feature engineering, enabling further application to prospective different population ages and pathologies.

## Methods

### Data collection and EEG recordings

#### Participants

Data were collected from 26 (7 male) adolescents (mean age = 15.04) with a recently sustained and clinically diagnosed concussion (mean days since insult = 20.15). A comparative group of 28 (5 male) participants (mean age = 19.3) acted as healthy controls, reporting no previous head injuries. All participants reported no neurological or auditory problems. The study was reviewed and approved by the Hamilton Integrated Research Ethics Board, Hamilton, Ontario, Canada. Prior to study participation, all participants provided informed consent in accordance with the ethical standards of the Declaration of Helsinki.

#### EEG stimuli and experimental conditions

ERPs were collected to a multi-deviant auditory oddball paradigm^10,45^. A 600-tone sequence was presented across two blocks of 300 each. Three deviant tones were presented pseudo-randomly in a continuous stream of standard tones. The standard tone was presented 492 times (82%) at 1000 Hz, 80 dB sound pressure level (SPL), and a duration of 50 ms. Each deviant was presented 36 times (6%) and differed from the standard tone in only one sound characteristic. The frequency deviant was 1200 Hz, the duration deviant was 100 ms, and the intensity deviant was 90 db SPL. Participants were tasked to respond using one button to the standard and another button to all deviants. Due to technical issues, data from the intensity deviant were discarded during analysis.

#### Procedure

Participants were seated facing a computer screen in a dimly-lit, sound-attenuated room. Auditory stimuli were controlled and sequenced using Presentation software (Neurobehavioural Inc.). Stimuli were presented using noise-cancelling insert earphones (Etymotic ER-1). Participants were instructed to respond to the stimuli as accurately as possible. The protocol was 10 minutes long and was the first of a series of other protocols not pertinent to the present study.

### EEG recording and preprocessing

Continuous EEG was recorded from 64 Ag/AgCl active electrodes (Biosemi ActiveTwo system) placed according to the extended 10/20 system using an elastic cap. Data were passed through an online bandpass filter of 0.01–100 Hz and referenced to the driven right leg. Data were digitized and saved at 512 Hz. Five external electrodes were recorded with the same settings. Three were placed on the mastoid processes and on the tip of the nose. The last two were placed above and over the outer canthus of the left eye to record eye movements. Stimuli markers were recorded and saved synchronously with the EEG data.

Data were processed offline using a 60 Hz notch and a 0.1–30 Hz (24 dB/oct) bandpass filters before re-referencing to the averaged mastoids. Artifacts were rejected manually using visual inspection followed by independent component analysis (ICA) decomposition. The two components found to correlate with horizontal eye movements and blinks were removed before recomputing sensor data. Trials with correct behavioural responses were segmented to 1200 ms intervals starting 200 ms before stimulus onset. Finally, segments were baseline corrected (−200 to 0 ms) and grouped into their respective experimental conditions before exporting the single trials. All EEG preprocessing was conducted using Brain Vision Analyzer (v2.01; Brain Products GmbH).

### Statistical analyses

Mixed effects analysis of variance (ANOVA) was used to examine the effects of Testing Date (2 levels: First and Second) and Recovery (2 levels: symptom resolution [SR] and no symptom resolution [NSR]) on the accuracies reported by TRODNet.

### Machine learning procedure

#### Input structure

The number of trials ${t}_{i}^{d}$, such that the superscript *d* indicates condition, extracted from each subject *i* was set to 36 to match the design’s maximum for each deviant condition. In the standard condition, 36 trials were sampled without replacement for each subject. In cases when rejected data reduced the number of a deviant’s trials below 36, bootstrapping was conducted to ensure ${t}_{i}^{d}=36$. The deep learning classifier concurrently processed a single trial of data from each condition as input observation $O\,\in \,{{\mathbb{R}}}^{N\times S}$ where *N* was number of EEG channels (*C*) × the number of conditions (*D*), and *S* was the number of samples in each segmented trial. Passed samples were restricted to the 50–700 ms window such that *S* = 332. *C* was 64 channels and *D* was 3 conditions, yielding *N* = 192. Before dataset split, there were ${T}_{main}={t}_{i}^{d}\times \#Subjects=1944$ unique observations across the two classes, as well as *T*_*longitudinal*_ = 684 longitudinal observations collected from concussed subjects on their second day of testing. We denote the main dataset tensor as $X\,\in \,{{\mathbb{R}}}^{{T}_{main}\times N\times S}$. All EEG data manipulation was conducted using the Python MNE package^46^.

#### Training and validation

Stratified 10-fold cross-validation was applied to estimate the generalization accuracy of the trained models (see Fig. 1C). *X* was split into *X*_*train*_ and *X*_*test*_ before standardizing both sets based on *X*_*train*_, removing the mean and scaling to unit variance for each feature. Observations from one subject were contained exclusively in either *X*_*train*_ or *X*_*test*_ to ensure no performance inflation due to subject-specific idiosyncrasies. The learner was batch-trained on *X*_*train*_ for 500 epochs where each epoch passed a batch of *B* = 160 randomly-picked observations from *X*_*train*_. The resultant model predicted the labels of each observation in *X*_*test*_ to produce the trial *accuracy*_*t*_. A thresholded version of *accuracy*_*t*_ evaluated the *accuracy*_*s*_ of all trials from a single subject. If more than 50% was achieved, the $accurac{y}_{s}^{i}$ for subject *i* tallied as correct. In instances where *X*_*test*_ contained one or more subjects that have undergone a second day of testing, the subjects’ second set of trials were evaluated in parallel to assess their follow-up test’s accuracy similar to what’s described above. This procedure was done to ensure an identical training-set for both testing dates as well as to eliminate the possibility of within-subject bias. No training was conducted on data collected at the second day of testing.

#### Neural network architecture and hyperparameters

Following the notion that a multi-channel EEG signal is the evolution of certain topographies across time^25,29^, TRODNet utilized convolutional layers to learn commonly occurring topographical maps (see Fig. 1B)^27,28^. The present architecture, based on an EEG ConvNet^28^ and EEGNet^27^, expanded to account for multiple conditions in the same input observation. Compared to EEGNet, TRODNet did not contain a convolutional layer that provided learned filtering settings, but split the depthwise convolution for each of the experimental conditions to extract topographical maps that best distinguish each condition. TRODNet corresponded in architecture to the shallow ConvNet^28^ with the addition of the by-condition split and by limiting the input to time-locked trials (see Fig. 1B). The network had four layers in total (in addition to input).

- *L*_*input*_: This describes the input layer. The input tensor is of size *B* × *N* × *S* and is reshaped to *B* × *N* × *S* × 1 before passing to the next layer.
- *L*_1_: The input tensor was split across three separate convolutional filters such that each was tasked with learning *M* = 5 maps that are specific to the condition. Kernel size was set to (64, 1). The output from each of the three sub-layers was of size *B* × 1 × *S* × *M*. The outputs were concatenated across the last dimension before passing to the next layer.
- *L*_2_: A maxpooling layer was applied with both a pool size and stride of (1, 10) and (1, 5), respectively.
- *L*_3_: Corresponded to a dense feed-forward layer of size 100.
- *L*_*output*_: The output layer acted as the label predictor with softmax activation to separate classes *concussed* and *control*.

All layers but *L*_*output*_ had a rectified linear activation unit (ReLU). *L*_2_ regularization was applied on all weights with *λ* = 0.25. The Adam optimizer was used during training with *α* = 5e − 4. Training for a single cross-validation iteration was stopped after 500 complete epochs. These hyperparameters were set to optimize a separate dataset^10,24^ collected using the same EEG/ERP protocol and were not modified throughout training. The code for training, testing, and visualization procedures is made readily accessible (see Data Availability section).

#### Model interpretation

The Deep Learning Important FeaTures (DeepLIFT)^47^ implementation using Shapley values^34^ was applied post-hoc on a model trained on all data to explain a model’s decision on single-subject averages. An overall estimate of all features’ influence on classification was calculated as the mean of the absolute SHAP values for all single-subject averages. The values were overlaid across the head to represent a 64-channel plot as commonly used in EEG/ERP studies. For visual clarity, each experimental condition was plotted independently.

## Data availability

The input set was imported and formatted using Python MNE^46^ package version 0.16.1 running on Python 3.5.2. Cross-validation and scaling were applied using scikit-learn 0.19.1^48^. Deep learning used Tensorflow^49^ (v1.8.0). All code is made available at https://github.com/boshra/TRODNet. Statistical analysis was conducted using R statistical software (v3.5.3) and the ez package (v4.4–0). Result storage, correlational plots, and feature importance visualizations were conducted using the pandas (v0.24.1), seaborn (v0.9.0), and Python MNE packages, respectively. The single-trial data used to train the models of this study are available upon request from the corresponding authors (J.F.C. and R.B.).
